# Pharmaco-Optogenetic Targeting of TRPC Activity Allows for Precise Control Over Mast Cell NFAT Signaling

**DOI:** 10.3389/fimmu.2020.613194

**Published:** 2020-12-18

**Authors:** Bernadett Bacsa, Annarita Graziani, Denis Krivic, Patrick Wiedner, Roland Malli, Thomas Rauter, Oleksandra Tiapko, Klaus Groschner

**Affiliations:** ^1^Gottfried-Schatz-Research-Center–Biophysics, Medical University of Graz, Graz, Austria; ^2^Gottfried-Schatz-Research-Center–Molecular Biology and Biochemistry, Medical University of Graz, Graz, Austria

**Keywords:** canonical transient receptor potential channels, mast cells, opto-chemical immunomodulation, NFAT nuclear translocation, photopharmacology, OptoBI-1

## Abstract

Canonical transient receptor potential (TRPC) channels are considered as elements of the immune cell Ca^2+^ handling machinery. We therefore hypothesized that TRPC photopharmacology may enable uniquely specific modulation of immune responses. Utilizing a recently established TRPC3/6/7 selective, photochromic benzimidazole agonist OptoBI-1, we set out to test this concept for mast cell NFAT signaling. RBL-2H3 mast cells were found to express TRPC3 and TRPC7 mRNA but lacked appreciable Ca^2+^/NFAT signaling in response to OptoBI-1 photocycling. Genetic modification of the cells by introduction of single recombinant TRPC isoforms revealed that exclusively TRPC6 expression generated OptoBI-1 sensitivity suitable for opto-chemical control of NFAT1 activity. Expression of any of three benzimidazole-sensitive TRPC isoforms (TRPC3/6/7) reconstituted plasma membrane TRPC conductances in RBL cells, and expression of TRPC6 or TRPC7 enabled light-mediated generation of temporally defined Ca^2+^ signaling patterns. Nonetheless, only cells overexpressing TRPC6 retained essentially low basal levels of NFAT activity and displayed rapid and efficient NFAT nuclear translocation upon OptoBI-1 photocycling. Hence, genetic modification of the mast cells’ TRPC expression pattern by the introduction of TRPC6 enables highly specific opto-chemical control over Ca^2+^ transcription coupling in these immune cells.

## Introduction

Photopharmacology and optogenetics have emerged as experimental strategies that allow for exceptionally precise interference with tissue functions (1, 2). These technologies have proven particularly successful in elucidating basic principles of communication within complex signaling networks and are suggested as a prospective basis for light-mediated computer-cell/tissue interfaces in the context of synthetic biology (3). So far optogenetic and chemo-optogenetic have unequivocally made significant contributions to our understanding of neuronal circuits and provided important insights into the complex orchestration of immune reactions. Moreover, the feasibility of optogenetic manipulation of immune responses has repeatedly been demonstrated in a therapeutic context (4–7).

We have recently developed a new photopharmacological tool that allows for specific, light-assisted control over TRPC3/6/7 conductances in native tissues (8). Here we set out to test the suitability of this new approach for modulation of immune responses, using the well-characterized RBL-2H3 mast cell model. Mast cells are tissue-resident and confer innate and adaptive immune reactions, thereby playing a pivotal role in allergic disorders, cancer, and autoimmune diseases (9). Mast cells mediate IgE-dependent allergic reactions by release of inflammatory mediators *via* degranulation, production of inflammatory lipids, and production of cytokines (10) and have long been recognized as a critical component of the tumor microenvironment in a number of cancer types (11–13). Based on the complex and essentially two-faced function of these tumor resident immune cells (12), therapeutic targeting of these cells requires uniquely specific approaches. High precision, local modulation of tumor-resident immune cells might represent a novel strategy for adjuvant immunotherapy in cancers (13). One approach to achieve sufficient specificity of immunomodulation is based on the finding that Ca^2+^ downstream signaling is strictly dependent on temporal signaling features (14, 15). Hence, the controlled sculpturing of immune cell Ca^2+^ signals is expected to enable control over immune responses in a uniquely specific manner. So far, the therapeutic modulation of immune cell functions by light has focused mainly on the major player in immune cell Ca^2+^ handling, the STIM/Orai Ca^2+^ entry complex (7). Nonetheless, other Ca^2+^ signaling elements may similarly serve as suitable targets for optical approaches. Although many other plasma membrane transporters and channels, including K^+^ channels and TRP channels are reportedly critical for immune cell activation (16–18), these molecular players have so far not been considered and tested as targets.

For canonical transient receptor potential (TRPC) channels a contribution to Ca^2+^ signaling in immune cells has repeatedly been suggested (19). While several studies provide evidence for a contribution of TRPC1 and TRPC5 proteins to store-operated calcium signaling in rat (RBL-2H3) and mouse bone marrow-derived mast cells (BMMC), the exact function of TRPC isoforms in mast cells remains largely elusive (20–22). Interestingly, TRPC3/6/7 protein complexes were found to interact with fyn kinase during Fc*ϵ*RI-mediated mast cell activation (23). However, a more recent study argues against the contribution of TRPC3/6 channels to Fc*ϵ*RI-mediated Ca^2+^ signaling in primary human lung mast cells as well as in LAD2 human mast cells, suggesting exclusively Orai but not TRPC may be considered as a target for the control of mast cells in allergic disease (24). Nonetheless, in clear contrast to a lack of linkage to FcϵRI stimulation, TRPC1, TRPC4, and TRPC6 proteins have been shown to confer downstream signaling in Mrgprb2-mediated mast cell activation of murine peritoneal mast cells (25). Thus, the efficacy and specificity of TRPC generated Ca^2+^ signals in terms of their coupling to downstream effectors might be strictly dependent on the mechanism and mode of activation.

In the present study, we explored the functional consequences of direct, lipid-independent control of TRPC3/6/7 conductances in RBL-2H3 mast cells by a new photochromic benzimidazole agonist (OptoBI-1). We report that overexpression of the TRPC6 isoform, and thus specific modification of the TRPC expression pattern of RBL-2H3 cells, allows for efficient and temporally precise control over NFAT1 signaling by light. Our results provide the first proof of concept for efficient chemo-genetic targeting of mast cell Ca^2+^ signaling and transcriptional regulation based on TRPC photopharmacology.

## Methods

### Reagents and Construct

All reagents used were of molecular biology grade, purchased from Sigma-Aldrich Handels GmbH unless specified otherwise (Vienna, Austria). CMV-R-GECO1.2 construct (#45494) was obtained from Addgene. The following constructs were generated: YFP-TRPC3, CFP-TRPC3, YFP-TRPC6, CFP-TRPC6-hTRPC3 (Q13507-3) and hTRPC6 (Q9Y210-1) genes were cloned into peYFP-C1 and peCFP-C1 vectors using EcoRI/XbaI cloning sites. The mCherry-NFAT1 was prepared by replacing CFP by mCherry in a CFP-NFAT (in peCFP-C1 vector) construct. The TRPC7-mseCFP construct (Q9WVC5-1 cloned into pCI-neo vector) was kindly provided by Prof. Yasuo Mori (Kyoto University, Japan). OptoBI-1 was synthesized at Bio-Techne (Bristol, GB).

### Cell Culture and Genetic Manipulations

All experiments were performed in rat basophilic leukemia cells (RBL-2H3) cells, and no special ethical considerations were applied. Cells were cultured in Dulbecco’s Modified Eagle Medium (DMEM, Invitrogen) supplemented with 10% fetal bovine serum (FBS), streptomycin (100 μg/ml), penicillin (100 U/ml), L-glutamine (2 mmol/L), and HEPES (10 mmol/L). Cells were maintained in an incubator at 37°C, 5% CO_2_. Plasmids were delivered into RBL-2H3 cells by electroporation in a 4-mm electroporation cuvette (Bio-Rad, US), by adding plasmid (5 μg mCherry-NFAT1 and R-GECO; 20 μg TRPC constructs) to 4 × 10^6^ cells in 400 μl Opti-MEM^®^ and applying 200 V and 950 μF in a Gene Pulser II (Bio-Rad, UK). After electroporation cells were transferred from the cuvette onto coverslips in a 35 mm dish containing cell culture medium. Experiments were performed 16–24 h after electroporation.

### RNA Isolation and Quantitative Real-Time PCR

RNA was isolated from cell lysates using EXTRACTME TOTAL RNA KIT with 1% *ß*-Mercaptoethanol (Blirt) and reverse transcribed using a high-capacity cDNA reverse transcription kit (Applied Biosystems, Foster City, CA, USA) in a thermal cycler (Bio-Rad) according to the manufacturer’s protocol. qPCR was performed using GoTaq^®^ qPCR and RT-qPCR Mix (Promega) in a LightCycler C1000 Thermal Touch, Thermal Cycler CFX96™ Real Time System (Bio Rad). Relative expression of the target gene was normalized to rat *ß-Actin* as a reference gene. Specific Primers used for rat Trpc genes are listed below.

**Table:** 

CDS TRPCs	Accession number	Orientation	Primer sequence (5’-3’)
rTrpC1	NM_053558.1	Forward Reverse	AGGTGACTTGAACATAAATTGCGT TCCATAAGTTTCTGACAACCGT
rTrpC2	NM_022638.3	Forward Reverse	ATCCCCTTTCGCCCAACTG ATCGGAGCCTGATTCCAGCAG
rTrpC3	NM_021771.2	Forward Reverse	GACGCAGTACGGCAACATCC ACCTCCAGATGCTCATTGCC
rTrpC4	NM_080396.1	Forward Reverse	AAACGAAATGTCAACGCCCC TCTCGGCTTCCTCCAGAGAT
rTrpC5	NM_080898.2	Forward Reverse	CATCGAGATGACCACAGCGA GGGAAGCCATCGTACCACAA
rTrpC6	NM_053559.1	Forward Reverse	GTGAACGAAGGGGAGCTGAA GCGGCTTTCCTCTTGTTTCG
rTrpC7	NM_001191691.2	Forward Reverse	GGGGTCCTGCCTACATGTTC CCCATGTAGTCCACGCAGTT
rActin		Forward Reverse	CGATATCGCTGCGCTCGT ATACCCACCATCACACACCCTG
rGapdh	NM_017008.4	Forward Reverse	CCTTCTCTTGTGACAAAGTGGACAT GCTTCCCATTCTCAGCCTTGA

### Electrophysiology

Whole-cell electrophysiology was performed at room temperature. RBL-2H3 cells were seeded on coverslips 24 h prior to the experiments. Coverslips were mounted in a cell bath on an inverted Axiovert 200 microscope (Zeiss). YFP-TRPC3, YFP-TRPC6 and TRPC7-CFP transfected cells were identified by their green and blue fluorescence when illuminated at 500 nm or 436 nm using Oligochrome light source (FEI, Germany). Whole-cell measurements were performed using an Axopatch 200B amplifier (Molecular Devices) connected with a Digidata-1440A Digitizer (Axon Instruments). Signals were low-pass filtered at 2 kHz and digitized with 8 kHz. The application of linear voltage-ramp protocols ranging from −130 to +80 mV (holding potential 0 mV) was controlled by Clampex 11.0 (Axon Instruments) software. Currents at −90 and +70 mV were plotted against time and normalized by capacitance. Maximal current–voltage relationships from −130 to +80 mV were subtracted and normalized by capacitance. Illumination protocol for photo-pharmacological measurements was applied as for Ca^2+^ imaging. Patch pipettes were pulled from thin-wall filament glass capillaries (Harvard Apparatus, W3 30-0068), using a Sutter Instruments P1000 puller and BOX filaments FB245B to a resistance of 2–4 MΩ. Cells were kept in Tyrode solution during the experiments. Pipette solution contained (in mM): 120 cesium methanesulfonate, 20 CsCl, 15 HEPES, 5 MgCl2, 3 EGTA, titrated to pH 7.3 with CsOH. The osmolarity of all solutions was between 290 and 315 mOsm.

### [Ca^2+^]_i_ Imaging

Changes in intracellular Ca^2+^ ([Ca^2+^]_i_) were monitored using red-shifted genetically encoded Ca^2+^ sensor (R-GECO1.2). Briefly, RBL-2H3 cells overexpressing R-GECO with YFP-TRPC3, YFP-TRPC6 or TRPC7-CFP, were washed twice with experimental buffer (EB composition in mM: 140 NaCl, 5 KCl, 1 MgCl_2_, 10 HEPES, 10 glucose and 1 CaCl_2_ (pH 7.4, adjusted with NaOH). Coverslips were mounted in a cell bath containing EB and 10 µM OptoBI-1 on an inverted microscope (Olympus IX71, Germany) with 40 × 1.3 N.A. oil-immersion objective. Cells were excited to follow the R-GECO signal using 577/25 nm filters *via* TILL Oligochrome light source (FEI, Germany) and fluorescent images were captured every second at 632 nm (using 632/60 nm emission filter, Chroma Technology, VT, USA) with an ORCA-03G digital CCD camera (Hamamatsu, Japan) using Live Acquisition 2.6 software (FEI, Germany). The *cis* isomerization of OptoBI-1 compound was triggered by exposure to 10 s illumination period at 365 nm, and subsequent reversal of OptoBI-1 to *trans* conformation was achieved with 430 nm light exposure to 10 s period. The cycling of UV (365 nm; violet) and blue light (430 nm; blue) illuminations were repeated three times. The interval between each illumination cycle was 60 s. All experiments were performed at room temperature. Detailed photocycling protocol is described in (26).

### NFAT Nuclear Translocation

Translocation of NFAT in mCherry-NFAT1 used as a control and YFP-TRPC3, YFP-TRPC6 and TRPC7-CFP overexpressed in RBL-2H3 cells was observed using an inverted microscope (Olympus IX71, Germany) equipped with a 40 × 1.3 NA oil immersion objective. During the recordings using Live Acquisition v2.6 software (TILL Photonics FEI Company, Gräfelfing Germany), the excitation of mCherry was achieved using 577/25 nm filter and fluorescent images were captured every 2 s at 632 nm (using 632/60 nm emission filter Chroma Technology, VT, USA) with an ORCA-05G digital CCD camera (Hamamatsu, Herrsching am Ammersee, Germany). ImageJ 1.51n software was used to measure the fluorescence intensity in the nucleus and cytoplasm before and after stimulation with 10 µM OptoBI-1. These values (nucleus/cytosol) were then plotted using the SigmaPlot 14.1 software (Systat Software Inc.). The translocation of mCherry-NFAT1 in RBL-2H3 cells was observed for 12 min.

### Fluorescence Imaging

Images from RBL-2H3 cells overexpressing CFP-TRPC3, CFP-TRPC6 and TRPC7-CFP were taken with the Zeiss array confocal laser scanning microscope (ACLSM, Zeiss Axiovert 200 M) using a 100×/1.45 oil immersion objective (Zeiss Microsystems, Jena, Germany). Illumination at 445 nm with an argon laser system (series 543, CVI Melles Griot, CA, USA) and emissions collected with a CCD camera (CoolSnap HQ2, Photometrics, Tucson, Arizona, USA). Image analysis was performed in ImageJ open source imaging analysis software (https://fiji.sc/).

### Statistical Analysis

Data analyses and graphical display were performed using Clampfit 11 (Axon Instruments) and SigmaPlot 14.1 (Systat Software Inc.). Data are presented as mean values ± S.E.M. Primarily, a Shapiro–Wilk test was conducted to test for normality of the value distribution. Whenever a normal distribution criterion was met, we used ANOVA to analyze the statistical significance. In general, differences were considered significant at p < 0.05 and indicated for individual comparisons in figures (* p < 0.05, ** p < 0.01, *** p < 0.001).

## Results

### Benzimidazole (OptoBI-1)-Mediated Control Over RBL-2H3 Mast Cell Ca^2+^ Signaling Requires Expression of Recombinant TRPC6 and TRPC7

**Figure 1:**
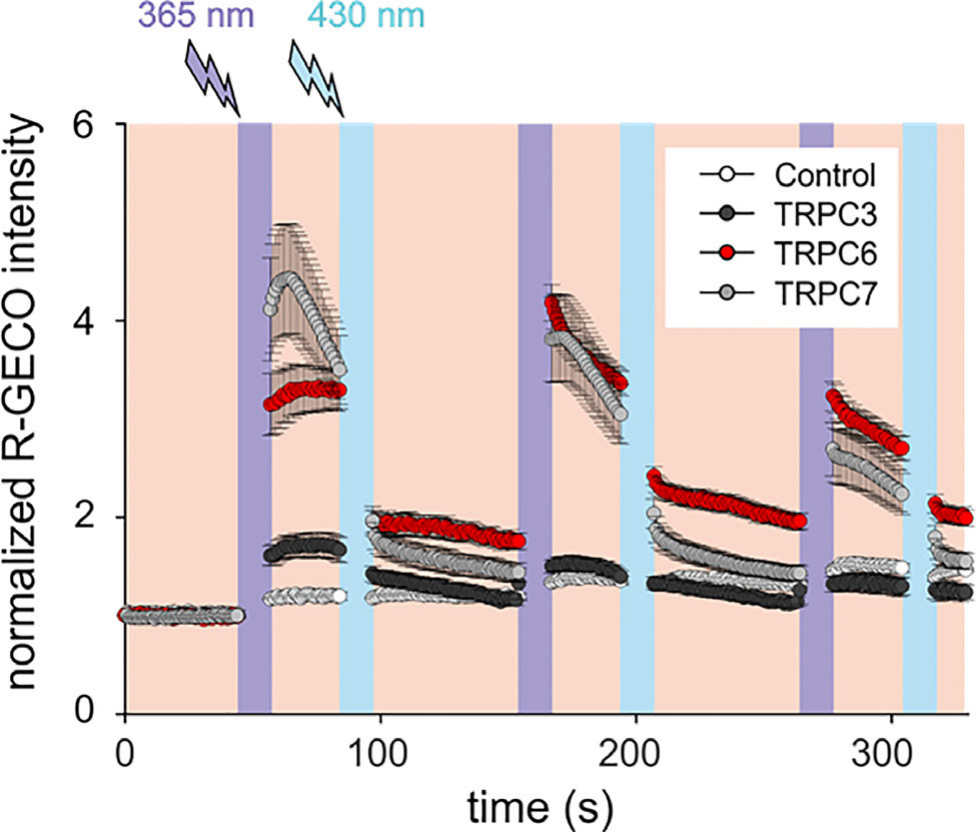
OptoBI-1 photocycling induced Ca^2+^ signaling triggered by overexpressed TRPC3, TRPC6 or TRPC7 channels in RBL-2H3 cells. Comparison of responses in cells overexpressing YFP-TRPC3, YFP-TRPC6 or TRPC7-CFP together with R-GECO as a Ca^2+^ reporter. Cells expressing R-GECO was only used as a control. Time courses (mean ± SEM) of R-GECO fluorescence intensity (red, 577 nm) during photoactivation of TRPC3 (black, n = 8 cells), TRPC6 (red, n = 23 cells), TRPC7 (gray, n = 21 cells), and controls (white, n = 10 cells). *Cis-trans* OptoBI-1 photocycling was repeated three times, by illuminating cells with UV (365 nm, 10 s; violet) followed by blue light (430 nm, 10 s, blue).

Mammalian mast cells have been shown to express an array of TRPC gene products (27). Our initial experiments to explore the sensitivity of mast cell Ca^2+^ signaling to a new photochromic TRPC ligand (OptoBI-1, **Figure 1**), clearly indicated that endogenous expression of benzimidazole TRPC target channels (8) is below the threshold for effective photopharmacological intervention in RBL-2H3 mast cells. Nonetheless, our analysis of the expression profile for TRPC subtypes in RBL-2H3 cells revealed that besides TRPC1 and TRPC4 also two potential benzimidazole targets, *i.e.* TRPC3 and TRPC7, were expressed at the mRNA level, while TRPC6 expression was not detectable (**Supplementary Figure 1**). Consistent with the lack of Ca^2+^ signals generated in native RBL cells by OptoBI-1 photocycling (**Figure 1**; control), we failed to detect TRPC3 by immunoblotting. Unfortunately, a suitable antibody for TRPC7 detection is currently not available. Next, we attempted to reconstitute OptoBI-1 sensitive cation conductances in RBL-2H3 cells by overexpression of a single benzimidazole responsive TRPC proteins (TRPC3/6/7). Mast cells were genetically modified to express single OptoBI-1 target channels in combination with R-GECO as a reporter for cytosolic Ca^2+^ changes (8, 26). The overexpression of single TRPC proteins allowed us to investigate the consequences of photoactivation for reconstitution of each TRPC channel subtype in our mast cell model. Genetically modified RBL-2H3 cells were exposed to photocycling of OptoBI-1 to induce defined pattern of transient rises in cytosolic Ca^2+^ as shown in **Figure 1**. These changes were modest in cells overexpressing TRPC3 but profound in cells expressing TRPC6 or TRPC7. OptoBI-1-induced peak values of normalized R-GECO fluorescence in TRPC6 (3.29 ± 0.26; n = 23) and TRPC7 (4.4 ± 0.56; n = 21) overexpressing RBL cells remain below the maximum SOCE-mediated signals achieved by thapsigargin (1 µM, 6.2 ± 0.14; n = 7), given as a reference stimulus. Of note, both “on” and “off” kinetic of light-controlled Ca^2+^ changes were essentially fast allowing for precise control over signal frequency and duration. For TRPC6 the reversal of cellular Ca^2+^ levels upon channel deactivation was incomplete and showed a tendency to remain elevated above control levels between consecutive photocycles. Importantly, TRPC6 as well as TRPC7 expression enabled the light-mediated generation of temporally defined Ca^2+^ signaling pattern, with TRPC7 producing the largest UV light-induced rise in cytoplasmic Ca^2+^ during the first illumination cycle as well as the most prominent desensitization during consecutive photocyling.

### Recombinant TRPC3, TRPC6, and TRPC7 Are Similarly Targeted to the Plasma Membrane but Produce Divergent Levels of OptoBI-1-Sensitive Cation Conductances

**Figure 2:**
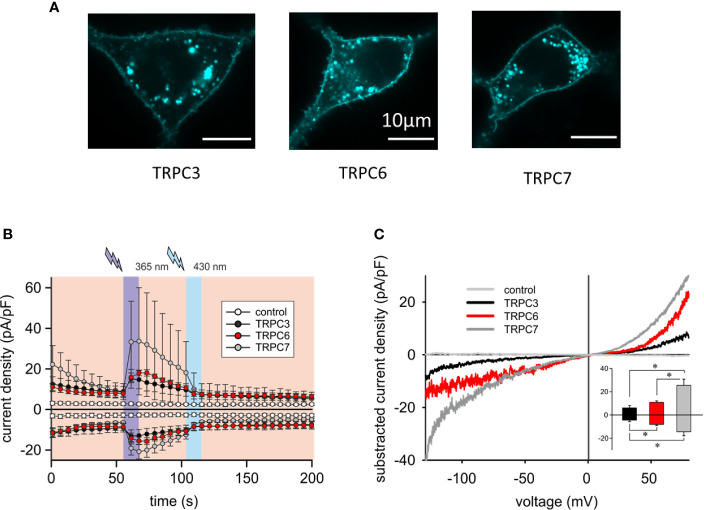
Overexpressed TRPC3/6/7 proteins are targeted to the plasma membrane of RBL-2H3 cells and generate cation conductances with divergent efficiency. **(A)** Representative epifluorescence images of RBL-2H3 cells expressing CFP-TRPC3 (n = 57), CFP-TRPC6 (n = 26) and TRPC7-CFP (n = 45), respectively. Scale bar = 10 µm. **(B)** Time courses of control (sham transfected, white, n = 6 cells), YFP-TRPC3 (black, n = 6 cells), YFP-TRPC6 (red, n = 8 cells) and TRPC7-CFP (grey, n = 8 cells) conductances recorded at −90 to +70 mV during repetitive photoconversion of OptoBI-1. Light illumination cycling is indicated as violet (365 nm) and blue (430 nm). **(C)** Representative net I–V relations (*I*_max_ − *I*_basal_) of OptoBI-1 induced currents in YFP-TRPC3 (black), YFP-TRPC6 (red), TRPC7-CFP (dark grey) transfected RBL-2H3 cells applying voltage-ramp protocols. Insert: Current density of net, maximum responses obtained at −90 to +70 mV (mean ± SEM). Statistical significance was tested by two tailed t-test (normally distributed values) or Mann–Whitney tests (non-normally distributed values), *p < 0.05, if not indicated differences are not significant (p > 0.05).

To better understand the mechanistic basis of the observed differences in reconstitution of OptoBI-1 sensitive Ca^2+^ signaling by the individual TRPC isoforms in RBL-2H3 cells, we continued with examination of the cellular localization and characterization of the membrane conductances generated with recombinant TRPC3/6/7. As shown in **Figure 2A** and **Supplementary Figure 2A**, recombinant TRPC channels fused to fluorescent markers (CFP or YFP) were similarly targeted to the plasma membrane. All cells expressing a TRPC fusion construct exhibited clear plasma membrane localized fluorescence. Of note, modification of RBL-2H3 cells to overexpress single TRPC-CFP fusion constructs did not affect cell morphology (**Figure 2A**) or promoted signs of degranulation. The OptoBI-1-induced TRPC conductances were quantified by electrophysiology applying the same OptoBI-1 photocycling protocol as in experiments measuring cytoplasmic Ca^2+^ rises (**Figure 2B**). All three TRPC isoforms reconstituted cation conductances with features consistent with those recently described for OptoBI-1-activated recombinant channels in HEK293 cells (8). These features included the I–V characteristics (**Figure 2C**) and current inactivation/desensitization, consistent with previous reports on benzimidazole agonists as well as photochromic actuators (28, 29). In full agreement with the OptoBI-1-induced Ca^2+^signaling, recombinant TRPC3 produced the smallest conductance, while TRPC7 expressing cells showed the largest current density after photoactivation (**Figure 2C**). Hence, the superior impact of TRPC6 and TRPC7 on mast cell Ca^2+^ homeostasis corresponds to larger cation conductances generated by these isoforms in RBL-2H3 cells combined with the reported higher Ca^2+^ permeability as compared to TRPC3 (30).

### Genetic Modification of RBL-2H3 Cells to Overexpress TRPC6 Enables Control Over Cellular NFAT Activity by Light

**Figure 3:**
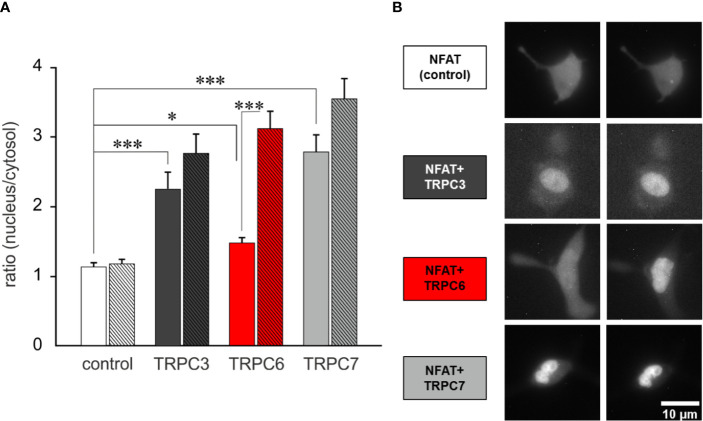
TRPC6 expression enables OptoBI-1-mediated control of NFAT1 activity in RBL-2H3 cells by light. **(A)** NFAT1 nuclear translocation in RBL-2H3 cells overexpressing either mCherry-NFAT1 only (control; white, n = 30) or mCherry-NFAT1 along with YFP-TRPC3 (black, n=17), YFP-TRPC6 (red, n = 31) or with TRPC7-CFP (gray, n = 21). Mean ± SEM nucleus/cytosol fluorescence intensity ratio. The translocation of mCherry-NFAT1 was monitored during OptoBI-1 photoconversion, applying three photocycles of illumination (three Flashes) as indicated. Statistical significance was tested by One-Way ANOVA (Holm–Sidak test for normally distributed values) or One-Way ANOVA for Ranks (Dunn’s test for non-normally distributed values), *p < 0.05, ***p < 0.001, if not indicated—not significant (p > 0.05). **(B)** Images of NFAT1 translocation in RBL-2H3 cells expressing mCherry-NFAT1 only (control; white) or coexpressing mCherry-NFAT1 and YFP-TRPC3 (black), YFP-TRPC6 (red) or TRPC7-CFP (grey) before (0 min) and after (12 min) three cycles of UV (365 nm, 10 s) and blue light (430 nm, 10 s) illuminations. Scale bar represents 5 µm.

In a next step we explored whether the generation of OptoBI-1 sensitivity in RBL-2H3 by overexpression of a single TRPC isoform is suitable to gain control over downstream Ca^2+^-dependent gene transcription. We set out to test the concept of opto-chemical control over NFAT1 activity by genetic modification of the mast cell’s TRPC expression pattern. NFAT1 nuclear translocation was recorded by expressing mCherry-NFAT1 fusion protein as a reporter, and applying the above-described protocol of repetitive photoactivation (three flashes of UV illumination). The extent of NFAT activation was quantified before and 12 min after initiation of the channel activation/deactivation cycles. Basal NFAT1 translocation was generally increased in all cells modified to overexpress TRPC channels. Nonetheless, this increase was modest with TRPC6 expression (**Figure 3A**) and repetitive, transient activation of the TRPC6 conductance by light resulted in a robust and highly significant NFAT1 nuclear translocation (**Figure 3** and **Supplementary Video 1**). By contrast cell expressing TRPC3 or TRPC7 displayed basal nuclear NFAT1 localization at levels comparable to that maximally achieved by TRPC6 activation. This phenomenon may be related to differences in basal channel activity, with an essentially low constitutive activity of TRPC6 (31), RBL cells expressing this channel subtype, displayed the lowest basal conductance measured immediately upon obtaining whole cell configuration in the absence of OptoBI-1 (**Supplementary Figure 3**). Importantly, only overexpression of TRPC6 channel in RBL-2H3 cells enabled precise control over mast cells Ca^2+^ transcription coupling by the photochromic benzimidazole OptoBI-1.

## Discussion

With the present study, we provide evidence for practicability of chemo/pharmaco-optogenetic modulation of immune cells function. This strategy is based on targeting overexpressed TRPC6 channels by the photochromic benzimidazole OptoBI-1. We report proof of this concept by demonstrating control over NFAT1 activation in RBL-2H3 by light.

RBL-2H3 cells were found to express TRPC proteins, specifically TRPC3 and TRPC7, which reportedly confer sensitivity to benzimidazole photopharmacology (8). However, this endogenous TRPC expression pattern was insufficient to generate significant cellular Ca^2+^ signals and NFAT1 translocation in response to OptoBI-1 photocycling. Lack of benzimidazole sensitivity of native RBL-2H3 mast cells may be explained by essentially low TRPC expression at the protein level. TRPC3 protein was indeed barely detectable by immunoblotting (not shown), while a test for TRPC7 protein expression was hindered by the lack of an appropriate antibody. Nonetheless, it appears reasonable to assume that the expression of endogenous benzimidazole target channels (TRPC3/6/7) is below the threshold for coupling to the Ca^2+^/CaN/NFAT pathway. Of note, in certain cellular settings TRPC3-generated Ca^2+^ signals failed to serve as an upstream trigger signal for NFAT activation (32). Nonetheless, the coupling of TRPC activity to downstream effectors may be strictly dependent on their mode of activation (33). In a recent study, we were able to demonstrate the linkage of recombinant TRPC channels to the calcineurin (CaN)/NFAT pathway in HEK293 cells (26). Expression of a TRPC3 gain-of-function mutant displaying enhanced benzimidazole sensitivity, was found to enable control of NFAT activity by OptoBI-1 (26). Consequently, we explored the option to achieve high precision control over mast cell NFAT signaling by combining photopharmacology and genetic modification. To do so, we altered mast cell TRPC expression by individual overexpression of benzimidazole-sensitive TRPC isoforms. Overexpression of recombinant TRPC3, TRPC6, or TRPC7 channels in RBL-2H3 cells reconstituted TRPC conductances with a clear order of efficacy with the largest OptoBI-1-induced current densities measured in TRPC7 expressing cells, whereas recombinant TRPC3 produced only a modest benzimidazole-sensitive conductance. All recombinant channel isoforms were found well targeted to the mast cell plasma membrane. The reason for the substantial difference observed upon reconstitution of TRPC3 and TRPC7 in RBL-2H3 cells remains unclear. Light-activated TRPC conductances displayed marked inactivation/desensitization as reported previously for benzimidazole agonists as well as lipid actuators (28, 29). The prominent inactivation observed for TRPC7, may in part be explained by the high current density, considering a current- and/or Ca^2+^-dependent mechanism. Consistent with the higher Ca^2+^ selectivity of TRPC6 and TRPC7 channels, surmounting that of TRPC3 (34), OptoBI-1-photocycling exerted a profound impact on mast cell Ca^2+^ levels when cells expressed either TRPC6 or TRPC7, but not with TRPC3 expression. Thus, genetic modification of mast cells to overexpress TRPC6 or TRPC7 channels generated OptoBI-1 sensitivity that allows temporal sculpturing of Ca^2+^ signals in these immune cells. Notably, membrane currents and cytosolic Ca^2+^ levels did not decline synchronously upon fast, light-induced deactivation as previously reported for TRPC3 mutants in the HEK293 expression system (26). Remarkably long-lasting Ca^2+^ elevations were triggered by repetitive TRPC6 activation. This phenomenon may be related to cellular localization of these channels relative to major Ca^2+^ extrusion systems. Since TRPC6 has repeatedly been found co-localized with NCX1 (35–37), it is tempting to speculate that Na^+^ loading during TRPC6 activation might counteract NCX1-mediated Ca^2+^ extrusion thereby generating tonic elevation of basal Ca^2+^ upon repetitive activation. Importantly, only the introduction of an expression pattern featuring TRPC6 as the prominent species, provided a basis for efficient optical control over NFAT activation in the mast cells. Of note, all three benzimidazole-sensitive isoforms were found to communicate with the CaN/NFAT pathway, albeit in a divergent manner. TRPC3 and TRPC7 overexpressing mast cells displayed significant constitutive levels of NFAT1 nuclear localization, indicating the generation of a tonic increase of NFAT dephosphorylation, due to Ca^2+^ signals that arise from the constitutive activity of the overexpressed TRPC channels. Constitutive inward currents generated by TRPC3 and TRPC7 expression may results in tonic NFAT1 activation and preclude further control of NFAT transcriptional activity by photocycling of OptoBI-1. In RBL cells overexpressing TRPC6, which displays essentially low constitutive activity (31), basal NFAT1 translocation remained close to controls, and short pulsatile activation-deactivation of TRPC6 channels by OptoBI-1 photocycling resulted in rapid and significant NFAT1 nuclear translocation (within 10 min—see video in **Supplementary Video 1**). It is important to note that OptoBI-1 activation of TRPC6 may generate channel features and local Ca^2+^ entry pattern different from those of TRPC channels activated in response to receptor-phospholipase C pathways. The complex cascades inevitably linked to receptor-mediated activation of TRPC channels have been reported to modify the coupling between TRPC and NFAT signaling in cardiac muscle (38). To this end, the molecular basis of the observed efficient linkage between OptoBI-1-activated TRPC6 conductances and NFAT1 nuclear translocation in RBL-2H3 remains elusive. Nonetheless, our results demonstrate the general ability of TRPC6 to serve an important function, which is executed mainly by STIM/Orai channels in immune cells. Importantly, NFAT1 nuclear translocation triggered by TRPC-mediated Ca^2+^ entry, both by constitutive channel activity as well as short, transient pulses of channel activation using OptoBI-1 photocycling is potentially devoid of eliciting excessive exocytosis/degranulation, and may thereby enable a more specific modulation of immune responses as expected from interventions targeting the STIM/Orai machinery. This concept may be of relevance for the development of highly specific interventions to modify immune reactions of mast cells but also other immune cells, which play a complex, dual role in cancer pathology. It remains to be clarified if the here described concept of immunomodulation by TRPC photopharmacology can be adopted for control of other immune cells and as a basis of therapeutic strategies.

## Data Availability Statement

The original contributions presented in the study are included in the article/**Supplementary Material**. Further inquiries can be directed to the corresponding author.

## Author Contributions

BB: Ca^2+^ imaging, manuscript writing, data analysis. AG: Ca^2+^ imaging, NFAT translocation, data analysis. DK: NFAT translocation, data analysis. PW: qPCR, primer design. TR: fluorescence microscopy. RM: experimental design. OT: patch-clamp, data analysis. KG: concept, experimental design, funding, and manuscript writing. All authors contributed to the article and approved the submitted version.

## Funding

This research was funded by FWF P3326 (KG). DK is a member of the PhD program (DK) “Metabolic and Cardiovascular Disease” [W1226].

## Conflict of Interest

The authors declare that the research was conducted in the absence of any commercial or financial relationships that could be construed as a potential conflict of interest.

The handling editor declared a past co-authorship with one of the authors, RM.
