# Supplementary material for: Pharmaco-Optogenetic Targeting of TRPC Activity Allows for Precise Control Over Mast Cell NFAT Signaling
Source: Front Immunol. 2020 Dec 18;11:613194. doi: 10.3389/fimmu.2020.613194 (PMC7775509; doi:10.3389/fimmu.2020.613194)
Supplement: Supplementary file 1 [file Table_1.docx]

Supplementary Material

#
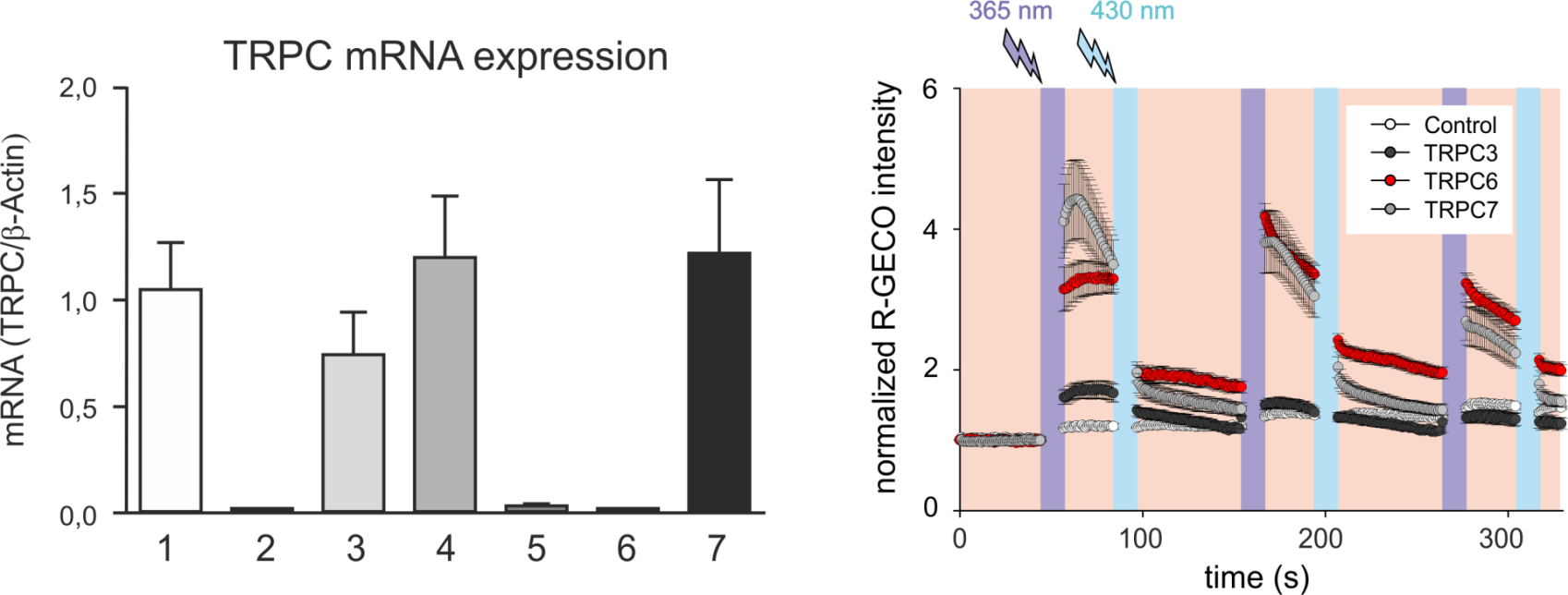


**Supplementary Figure 1.** Expression pattern of TRPC isoforms in RBL 2H3 cells. The expression of mRNA for seven members of the TRPC family was determined by RT-PCR.


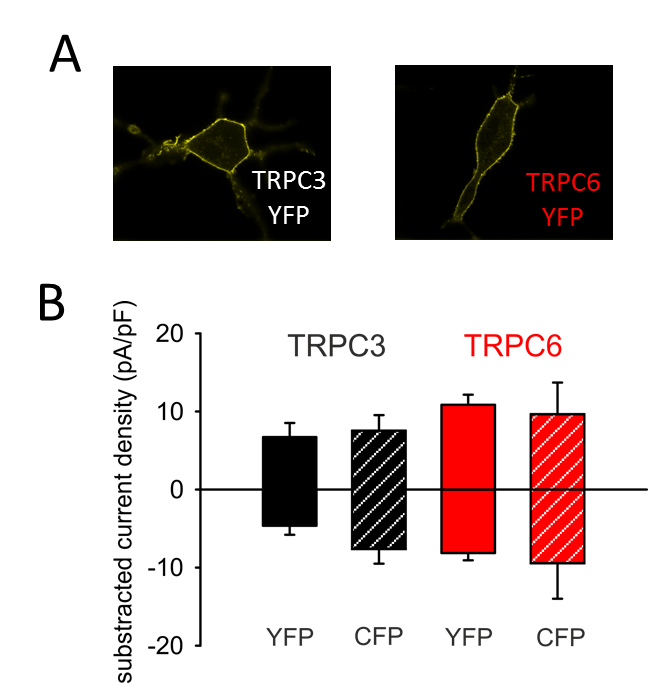


**Supplementary Figure 2. (A)** Representative epifluorescence images of RBL-2H3 cells expressing YFP-TRPC3 (n = 28) and YFP-TRPC6 (n = 28), respectively. Scale bar = 10 µm. (**B)** Current density of net, maximum responses obtained at -90 to +70 mV (mean ± SEM, n = 6-8 ). Statistical significance was tested by two tailed t-test (normally distributed values) or Mann–Whitney tests (non-normally distributed values), differences are not significant (p > 0.05).

.

**
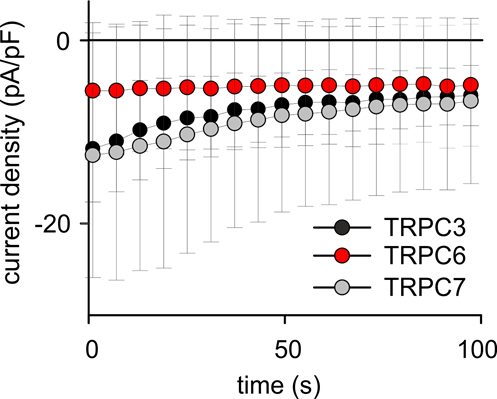
**

**Supplementary Figure 3.** Time courses of basal inward currents of YFP-TRPC3 (black, n = 5 cells), YFP-TRPC6 (red, n = 5 cells) and TRPC7-CFP (grey, n = 5 cells) overexpressed in RBL-2H3 cells. Conductance was recorded at -90 mV (mean ± SEM). Statistical significance of the decay of current densities within 100 s was tested by paired Student´s t test (normally distributed values), The decay was significant (p > 0.05) for TRPC3 and TRPC7 but not for TRPC6.

**Supplementary Video**: Light-induced TRPC6-mediated NFAT translocation, UV light was applied at frame 3.
